# Mouse genetic mosaic model for basal-like breast cancer reveals disruption of basal-myoepithelial architecture at premalignancy

**DOI:** 10.21203/rs.3.rs-10425660/v1

**Published:** 2026-07-29

**Authors:** Alexys T. Riddick, Xian Zhou, Hui Zong

**Affiliations:** University of Virginia

**Keywords:** basal-like breast cancer, premalignancy, basal-myoepithelial architecture, whole-mount imaging, mouse genetic mosaic model

## Abstract

In early cancer evolution, mutant cells must escape tissue-architectural constraints before progressing to malignancy. In the case of invasive breast cancers, loss of integrity of the wildtype basal-myoepithelial layer that wraps around premalignant luminal cells precedes the formation of an invasive tumor mass. The spatiotemporal details of these early events have remained elusive. Here, we used the Mosaic Analysis with Double Markers (MADM) mouse model of basal-like breast cancer to generate rare, GFP-labeled, premalignant luminal cells within an otherwise colorless, normal mouse, and pinpointed hyperalveolarization from the ducts as a key phase of progression, several months before malignant transformation. Using a whole-mount tissue clearing-staining-imaging workflow to examine the colorless basal-myoepithelial cell layer that surrounds GFP-labeled premalignant luminal cells, we observed a gradual disruption of the basal-myoepithelial layer during the hyperalveolarization phase. Increased cell size, altered cell morphology, and elevated proliferation of mutant cells occurred independently of basal disruption. In contrast, expression of the basal marker Keratin 14 in premalignant luminal cells, a feature that contributes to the “basal-like” nomenclature of this cancer, was strongly associated with basal disruption, suggesting that basal cell-mediated containment may suppress a critical transition in premalignant cell state. This work identifies disruption of the basal cell layer as an early hallmark of premalignant progression, supporting a chronology in which architectural remodeling of the mammary epithelium precedes malignant progression.

## Introduction

Progression from premalignancy to malignancy is a multistep process. In addition to acquiring cell-intrinsic genetic alterations, premalignant cells must overcome tissue-level constraints that suppress tumor formation. The interplay between molecular and cellular tumor-suppressive mechanisms creates a selective environment in which only a minority of premalignant cells ultimately progress to cancer. Detailed studies of these rare events may shed light on novel early detection and cancer prevention strategies.

For basal-like breast cancer (BLBC), loss of the molecular tumor suppressors *Brca1* and *Tp53* are early genetic events for initiation [1-6]. The cell of origin for BLBC resides within progenitors for the luminal epithelial compartment [1, 2, 7-9]. In a normal breast, ducts of luminal cells are surrounded by an outer layer of basal-myoepithelial cells that promote luminal cell differentiation and maturation during normal development [10-13]. During homeostasis, basal-myoepithelial cells also constrain luminal epithelial expansion through direct physical interactions [14, 15]. For example, the basal-specific adhesion molecule P-cadherin restricts luminal cell proliferation and alveolar differentiation, the loss of which results in excessive luminal proliferation and alveolar development in virgin mice [16]. Several other subsequent studies found that basal-myoepithelial cells suppress tumor progression and are lost in malignant BLBC [17-28]. Recently, studies have revealed tissue protection mechanisms in the mammary gland that suppress clonal growth [29]. However, when this basal cell-mediated containment is disrupted during premalignant progression remains unknown.

Investigating disruptions in precancerous tissue architecture in their native, undisturbed environment requires unequivocal visualization of rare premalignant cells *in vivo* and a comprehensive assessment of subtle changes in both premalignant lesions and their surrounding tissue architecture. For the former, we previously established a mouse model of BLBC using Mosaic Analysis with Double Markers (MADM-BLBC) [30], which generates rare GFP-labeled, *Brca1*,*Trp53*-null luminal cells within an otherwise normal mammary gland (**Supp 1A,B**). The rarity and permanent labeling of mutant cells permit high-resolution analysis of premalignant cells immediately following mutation acquisition. Previous studies with MADM-BLBC relied on two-dimensional imaging to characterize changes within mutant luminal cells; here, we adopted a tissue clearing and imaging protocol that is compatible with immunofluorescent staining and whole-mount, high-resolution imaging of the entire mammary gland [29, 31] (**Supp 1C**). Together, these approaches detected subtle changes in the tissue architecture and elucidated spatiotemporal events of altered basal architecture during premalignant progression.

**Figure 1:**
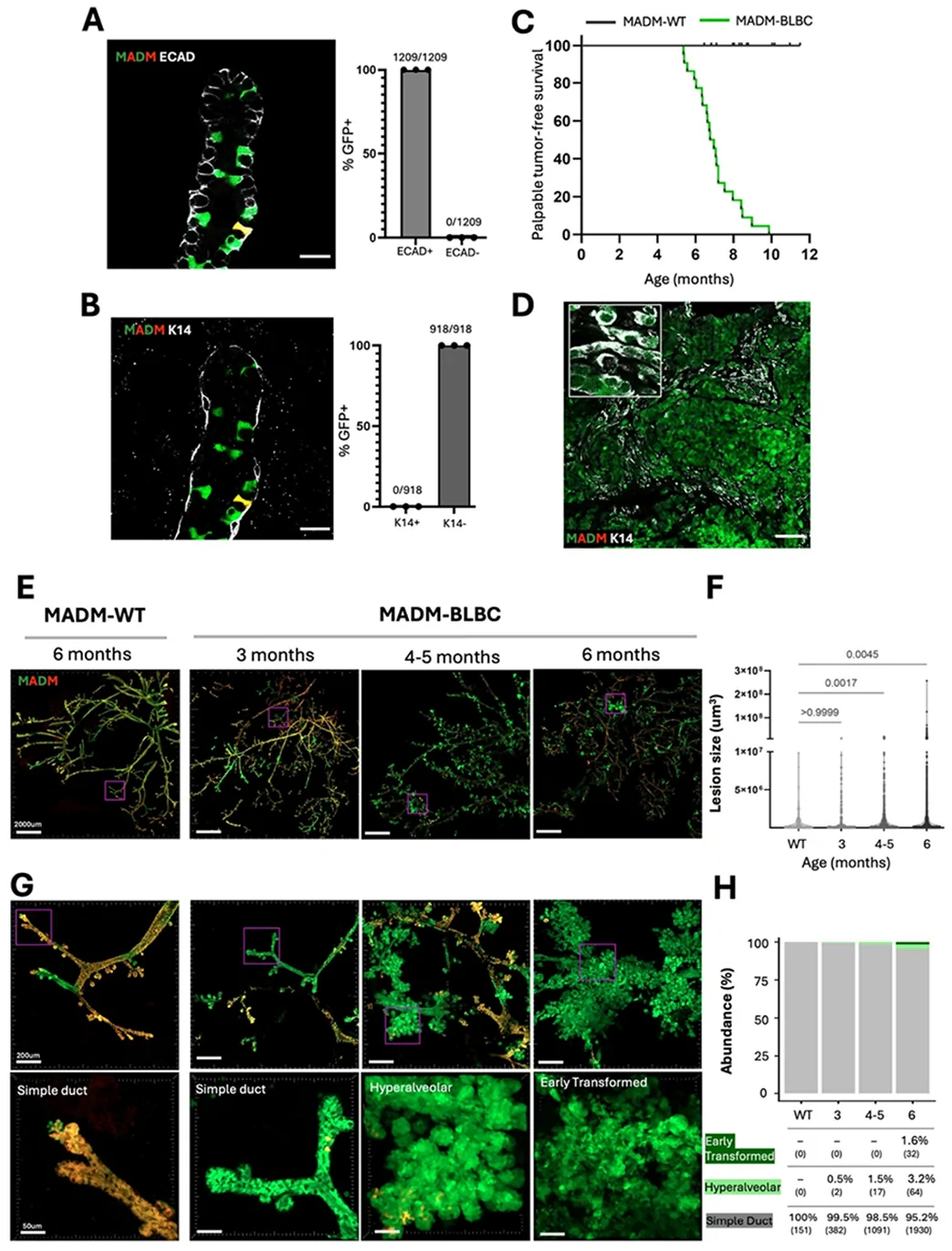
MADM-BLBC model reveals premalignant and early transformation phases prior to palpable tumor formation. **(A)** Representative image of 8-week-old MADM-BLBC mammary gland stained with E-cadherin (ECAD). Scale bar = 20um. Quantification across N=3 glands. **(B)** Representative image of mammary 8-week-old MADM-BLBC mammary gland stained with Keratin 14 (K14). Scale bar = 20um. Quantification across N=3 glands. **(C)** Palpable-tumor free survival of MADM-WT and MADM-BLBC mice. N= 14 MADM-WT mice; N=22 MADM-BLBC mice. **(D)** K14 staining in palpable GFP^+^ MADM-BLBC tumors. Scale bar = 100um. **(E)** Whole mount tiled images of representative mammary glands at multiple ages prior to palpable tumor formation. Scale: x = 13.23mm, y = 13.23mm , z = 90um. **(F)** Lesion size quantification from whole mount images at each age. N=3-5 mice per age. Kruskal Wallis test. **(G)** Top row: Higher magnification tiled images of inset magenta box from (E). Scale: x = 1.41mm, y = 1.41mm, z = 36um; Bottom row: Single field-of-view images of inset magenta box from top row. Scale: x = 319.45um, y = 319.45um, z = 36um. The associated phase is written on each image. **(H)** Abundance of premalignant phases at each age using data whole-mount image data from (F). Absolute number and percentage of each phase is written below. Thresholds for lesion size and morphology were set to define each phase: Simple duct (less than 1e+07um^3^; 0-5 alveolar buds), Hyperalveolar (between 1e+07um^3^ and 2e+07um^3^; >5 alveolar buds), Early transformed (greater than 2e+07um^3^, no identifiable ducts or alveolar buds).

**Supp. 1 (A)** Schematic of MADM’s Cre-loxP-mediated interchromosomal mitotic recombination for MADM, *Brca1,Trp53; MMTV-Cre* mice. From a colorless heterozygous cell, MMTV-Cre-mediated interchromosomal recombination during G2 produces one GFP^+^ *Brca1,Trp53*-mutant cell and one RFP^+^ wild-type cell after X segregation, when recombinant sister chromatids segregate into different daughter cells. Alternatively, Z segregation yields one colorless and one yellow (GFP^+^RFP^+^) heterozygous cell when recombinant sister chromatids segregate into the same daughter cell (top). More frequently, recombination occurs in colorless heterozygous cells without DNA replication (G0/G1), producing abundant yellow heterozygous cells (bottom). The MADM schematic is reproduced with permission from Zong et al. (2005). **(B)** Schematic representation of cells produced within MADM wildtype mice, without any mutations. **(C)** Schematic of whole mount mammary gland clearing-staining-imaging workflow. **(D)** Whole mount image from MADM-WT Fig. 1E, processed using Imaris to mask GFP^+^ lesions.

## Results

### MADM-BLBC model reveals premalignant and early transformation phases prior to palpable tumor formation.

We backcrossed the initially mixed background of MADM-BLBC to a pure FVB/NJ background, which improves tumor penetrance with a relatively consistent latency [32]. Although MMTV-Cre was shown to mediate recombination in both luminal and basal-myoepithelial cells in conventional mouse models when loxP sites are on the same chromosome (in *cis*), we found that MMTV-Cre mediated recombination in MADM occurred exclusively within the luminal compartment, likely because loxP sites are on different chromosomes (in *trans*) and thus require high Cre for recombination to occur [30, 33]. Importantly, during pubertal expansion, the G2-phase requirement for MADM labeling (**Supp 1A**) would restrict mutant cell generation to rapidly proliferating luminal progenitors, which are known to be the cell of origin for BLBC [1, 2, 8, 13, 34]. We validated recombination specificity by staining glands from 2-month-old MADM-BLBC mice for the luminal cell marker E-Cadherin (ECAD), and the basal cell marker Keratin 14 (K14), confirming that all GFP-labeled cells were ECAD positive and K14 negative (Figs. 1A,B). After confirming luminal cell origin, we assessed basal-like features of resulting tumors, including the invasive phenotype with the loss of basal-myoepithelial cells, and luminal cell plasticity by the gained expression of K14 [26, 27, 35-38]. Palpable tumors (arising at ~ 7 months) exhibited all these features in the GFP^+^ tumor mass (Figs. 1C,D). Together, these findings establish the beginning and endpoint of tumorigenesis in the MADM-BLBC model, providing the framework to investigate intermediate events during premalignant progression from luminal cell initiation to basal-like breast cancer formation.

To define the earliest phases of premalignant progression, we performed a time course analysis of the expansive patterns of GFP^+^ lesions prior to the formation of palpable tumors by using whole-mount three-dimensional imaging. We harvested MADM-BLBC mice between 3 and 6 months of age, along with MADM-WT mice harvested at 6 months as a control, and used a three dimensional clearing-staining-imaging workflow to quantify size differences between GFP^+^ lesions in MADM-WT and MADM-BLBC mice (**Supp 1C-D**). At low magnification, we observed a gross increase in GFP^+^ lesion abundance with age (Fig. 1E). Volumetric analysis of contiguous GFP^+^ lesions, presumably mutant clones, showed that, at 3 months, the size distribution of mutant lesions in MADM-BLBC mice were comparable in size to that of GFP^+^ wild-type ducts in MADM-WT mice (Fig. 1F). However, by 4–5 months, the volume of large lesions increased significantly and continued to grow by 6 months (Fig. 1F). Interestingly, large premalignancies represented only a small fraction of all GFP^+^ lesions: most remained small, suggesting that premalignant lesions are largely constrained.

To quantify morphological changes as lesions grow in size, we acquired high-resolution whole-mount images. 3-month-old MADM-BLBC and MADM-WT mice exhibited discrete stretches of GFP^+^ ducts with occasional alveolar budding from ducts, commonly seen during estrous cycling in virgin females (Fig. 1G) [29, 39], suggesting that mutant cell expansion initially is restricted within the mammary ducts [30]. At 4–5 months, GFP^+^ lesions developed numerous alveolar buds beyond those expected from estrous cycling, forming hyperalveolar lesions (Fig. 1G), which represent the first morphological distinction before substantial lesion expansion [9, 30]. By 6 months, small masses lacking recognizable ductal or alveolar architecture emerged, likely indicative of early transformation (Fig. 1G). As mice aged, simple duct lesions remained the predominant morphological phase at all ages, whereas rare hyperalveolar and early-transformed lesions became increasingly frequent in older MADM-BLBC mice (Fig. 1H). Together, these findings define distinct phases of premalignant progression and indicate that the hyperalveolar lesion represents a critical point at which a subset of mutant luminal cells escape normal growth constraints, prompting investigation into the intracellular and tissue-level changes that accompany this transition.

### Hallmark basal-like breast cancer features first present at the hyperalveolar premalignant phase.

**Figure 2:**
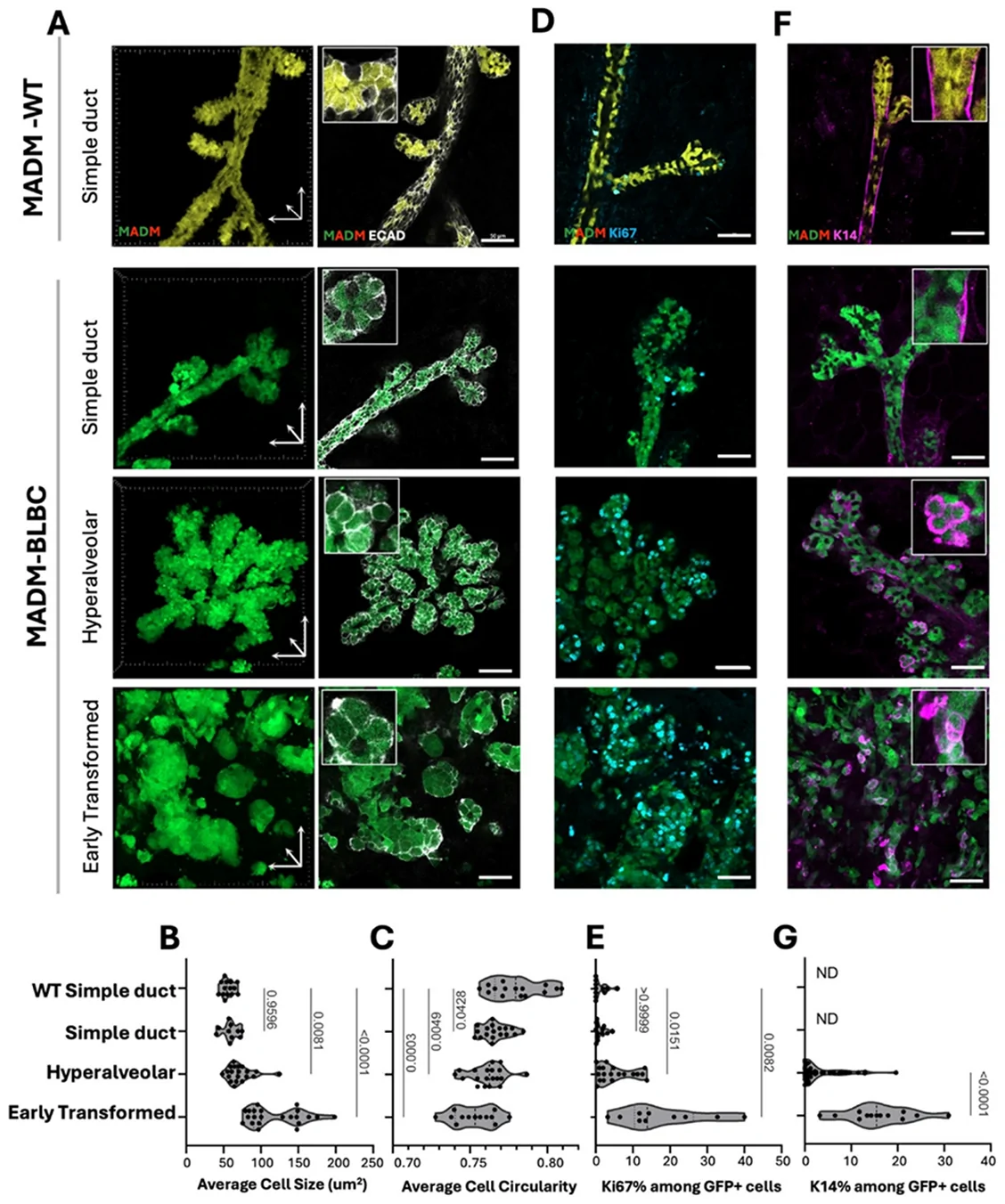
Hallmark basal-like breast cancer features first present at the hyperalveolar premalignant phase. **(A)** Left column: 3D projection of simple ducts, hyperalveolar and early transformed lesions. Scale: x = 319.45um, y = 319.45um, z = 36um; Right column: Optical cross-section with E-cadherin (ECAD) staining. Scale bar = 50um. Quantification in (B-C). **(B)** Quantification of the average cell size within each lesion. Cell size was averaged from over 1000 cells per lesion. N=3-5 mice per phase. Welch’s ANOVA with Dunnett’s T3 post-hoc test. **(C)** Quantification of the average cell circularity within each lesion. Cell circularity was averaged from over 1000 cells per lesion. N=3-5 mice per phase. Welch’s ANOVA with Dunnett’s T3 post-hoc test. **(D)** Optical cross-section of lesions with Ki67 staining. Scale bar = 50um. Quantification in (E). **(E)** Quantification of Ki67 percentage per lesion at each phase. N=3-5 mice per phase. Welch’s ANOVA with Dunnett’s T3 post-hoc test. **(F)** Optical cross-section of lesions with Keratin 14 (K14) staining. Scale bar = 50um. Quantification in (G). **(G)** Quantification of K14 percentage among GFP^+^ cells in lesions at each phase. N=3-5 mice per phase. ND = not detected. Welch’s t-test.

We next investigated the evolution of luminal cell-intrinsic features of basal-like tumors during premalignant progression, paying close attention to significant changes at the hyperalveolar phase. Since basal-like tumor cells have pleomorphic morphology and a high mitotic index [26], we used the luminal cell-specific membrane protein E-cadherin to assess cell size and circularity and Ki67 to detect proliferation in ductal, hyperalveolar, and early-transformed lesions. Individual cell size was comparable between MADM-WT (GFP^+^RFP^+^ appear yellow) and MADM-BLBC (GFP^+^ only) simple ducts, but increased significantly in hyperalveolar lesions and even further in early-transformed lesions (Figs. 2A,B). Cell circularity progressively decreased from the hyperalveolar phase onward, indicating increased irregularity of cell morphology (Figs. 2A,C). Lastly, we found that proliferation significantly increased at the hyperalveolar phase, and continued to progress at the early-transformed phase (Figs. 2D,E). Together, these data show that *Brca1,Trp53*-mutant cells overall become larger, more irregular in shape, and more proliferative at the hyperalveolar phase. The changes prompted us to ask whether hyperalveolarization is also accompanied by acquisition of the defining lineage features of basal-like tumors.

The most unique feature of basal-like tumors is the expression of basal cytokeratins on luminal cells, indicative of lineage plasticity that contributes to malignancy [2, 26, 35-38]. MADM-BLBC initiates from K14^−^ luminal cells and forms GFP^+^ tumors comprised of cells that are K14^+^ (Figs. 1B,D), providing an opportunity for us to determine when and where K14 misexpression starts during premalignant progression. At the hyperalveolar phase, we found that K14^+^GFP^+^ cells emerged sporadically and increased in abundance at the early-transformed phase (Figs. 2F,G). Thus, luminal cell misexpression of basal cytokeratin is an early event that occurs prior to overt transformation. Taken together, these data demonstrate that hallmark features of basal-like breast cancer—including increased proliferation, altered cell morphology, and acquisition of basal cytokeratin expression—first emerge heterogeneously at the hyperalveolar phase and increase into early transformation.

### Basal-myoepithelial architecture disruption emerges heterogeneously at the hyperalveolar phase.

**Figure 3:**
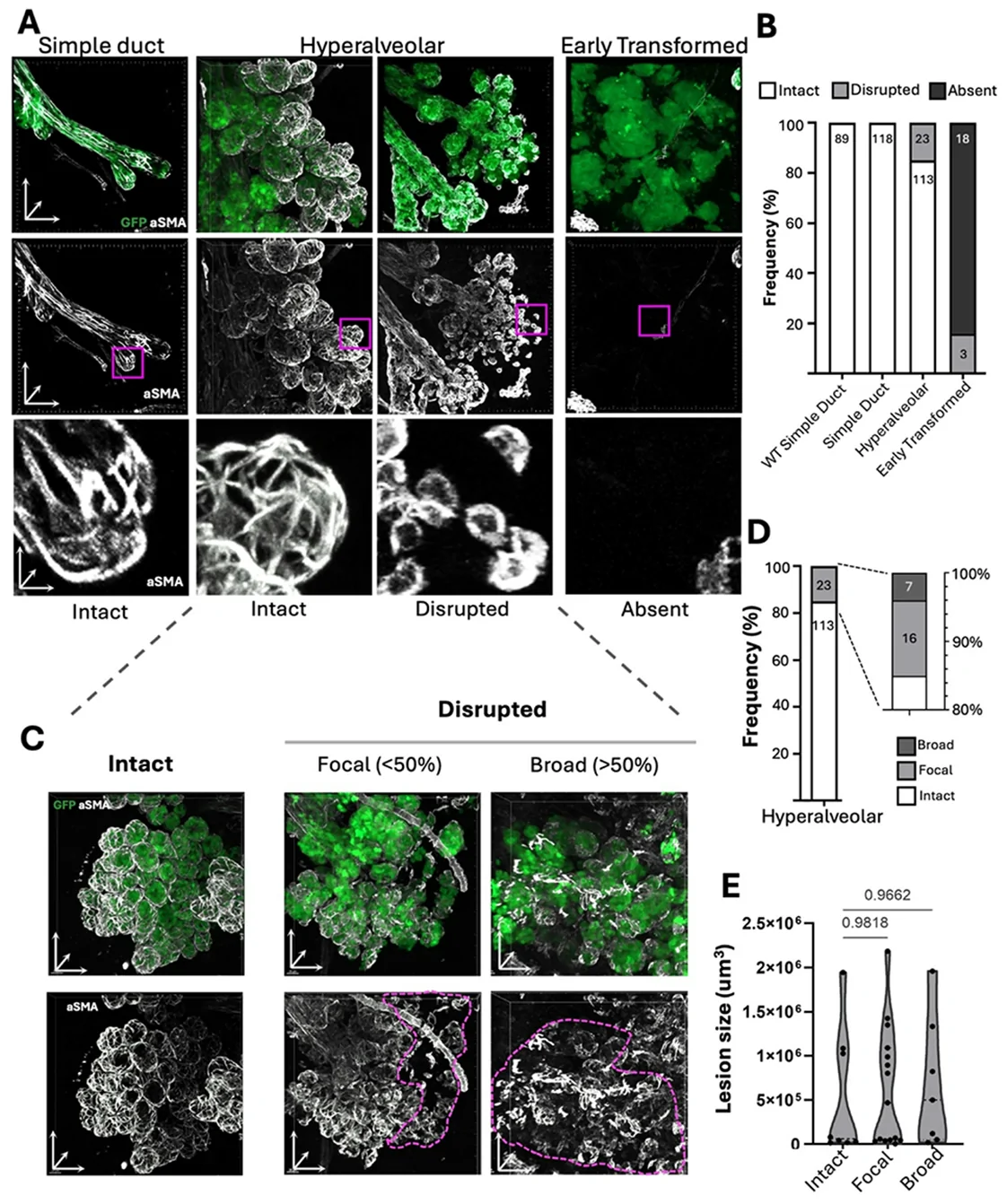
Basal-myoepithelial architecture disruption emerges heterogeneously at the hyperalveolar phase. **(A)** Top row: 3D projection of simple ducts, hyperalveolar and early transformed lesions with α -smooth muscle actin staining (αSMA). Scale: x = 319.45um, y = 319.45um, z = 36um; Middle row: 3D projection of αSMA staining. Scale: x = 319.45um, y = 319.45um, z = 36um; Bottom row: Zoom in of αSMA staining around one alveolar bud from magenta box in middle row. Scale: x = 50um, y = 50um, z = 36um. Quantification in (B). Note: αSMA labels endothelial vasculature in early transformed lesion. **(B)** Frequency of intact, disrupted or absent basal architecture at each phase measured from 15-100 lesions. N= 4-7 mice per phase. **(C)** Top row: 3D projection of hyperalveolar and early transformed lesions with degrees of basal architecture disruption. Scale: x = 319.45um, y = 319.45um, z = 36um; Bottom row: 3D projection of αSMA staining. Scale: x = 319.45um, y = 319.45um, z = 36um. Pink dashed line outlines disrupted region. Quantification in (D,E). **(D)** Frequency of intact, focal, or broad disruption in the hyperalveolar phase measured from ~150 lesions. N=4-7 mice. **(E)** Quantification of hyperalveolar lesion size with varying degrees of basal architecture disruption. Welch’s ANOVA with Dunnett’s T3 post-hoc test.

Since the hyperalveolar phase marks a critical cellular transition during premalignant progression, we next asked whether it is accompanied by changes in the surrounding basal-myoepithelial layer, a tissue-level tumor suppressor that normally constrains luminal cell behavior [18, 22, 40]. Whole-mount immunofluorescence for α-smooth muscle actin was used to visualize the entire basal-myoepithelial layer. As expected, an intact basal-myoepithelial layer composed of elongated cells aligned parallel to simple luminal ducts [41], and were absent in early transformed lesions (Figs. 3A,B). Interestingly, before overt transformation, a rare subset of hyperalveolar lesions exhibited disruption of the basal-myoepithelial layer, most frequently at the distal edge furthest from the main duct (Figs. 3A,B). These observations suggest that disruption of the basal-myoepithelial layer is not absolutely required for hyperalveolar lesion formation itself; rather it is likely a later event within a small subset of these lesions.

We next asked whether disruption of the basal-myoepithelial layer occurred abruptly or through a gradual process at the hyperalveolar phase. Our analysis of 23 hyperalveolar lesions with basal disruption revealed some partial disruption affecting less than half of the GFP^+^ lesion, and other broad disruptions affecting the majority of the lesion (Fig. 3C,D), suggesting that basal disruption likely develops gradually. One possible explanation for basal disruption is a simple consequence of luminal outgrowth beyond basal constraints. If true, broad disruptions would only occur in relatively large lesions. To test this possibility, we compared lesion size across different basal architectures and found that lesion size distribution did not differ among intact, focally disrupted and broadly disrupted lesions, suggesting that basal disruption is unlikely a simple consequence of physical forces generated by luminal outgrowth (Fig. 3E).

Together, these findings demonstrate that tissue-level containment is breached before overt malignancy. Basal-myoepithelial disruption emerges gradually, and stochastically, within a rare subset of hyperalveolar lesions independently of lesion size, likely representing a distinct biological transition during progression rather than a simple consequence of lesion growth.

### Basal architecture disruption correlates with luminal cell misexpression of Keratin 14.

**Figure 4:**
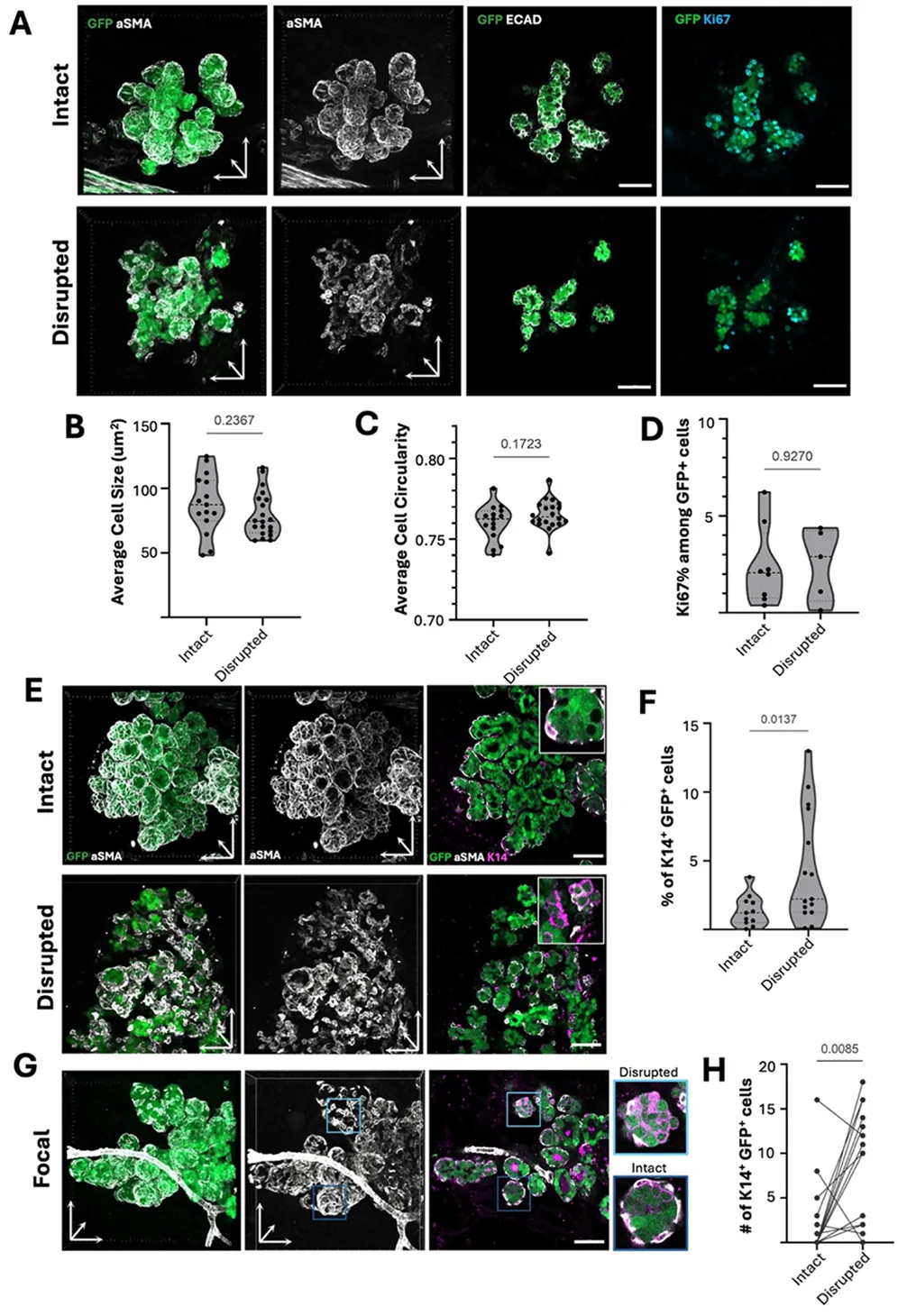
Basal architecture disruption correlates with luminal cell expression of keratin 14. **(A)** Far left: 3D projection of hyperalveolar lesions with GFP and a-smooth muscle actin (αSMA) staining. Scale: x = 319.45um, y = 319.45um, z = 36um; Middle left: 3D projection of αSMA staining. Scale: x = 319.45um, y = 319.45um, z = 36um; Middle right: Optical cross-section with E-cadherin (ECAD) staining. Scale bar = 50um. Quantification in (B-C); Far right: Optical cross-section with Ki67 staining. Scale bar = 50um. Quantification in (D). **(B)** Quantification of the average cell size within hyperalveolar lesions with intact or disrupted basal architecture. Cell size was averaged from over 1000 cells per lesion. N=3-5 mice. Welch’s t-test. **(C)** Quantification of the average cell circularity within hyperalveolar lesions with intact or disrupted basal architecture. Cell circularity was averaged from over 1000 cells per lesion. N=3-5 mice. Welch’s t-test. **(D)** Quantification of Ki67 percentage within hyperalveolar lesions with intact or disrupted basal architecture, taken across 3-5 mice. Welch’s t-test. **(E)** Left: 3D projection of hyperalveolar lesions with GFP and αSMA staining. Scale: x = 319.45um, y = 319.45um, z = 36um; Middle: 3D projection of αSMA staining. Scale: x = 319.45um, y = 319.45um, z = 36um; Right: Optical cross-section with Keratin 14 (K14) staining. Scale bar = 50um. Of note: K14 still labels basal-myoepithelial cells and colocalizes with αSMA in the intact lesion; there are no K14^+^GFP^+^ luminal cells in this region. Quantification in (F). **(F)** Quantification of K14 percentage among GFP^+^ cells within hyperalveolar lesions with intact or disrupted basal architecture. N=3-5 mice. Welch’s t-test. **(G)** Left: 3D projection of hyperalveolar lesions with focal basal disruption. Scale: x = 319.45um, y = 319.45um, z = 36um; Middle: 3D projection of αSMA staining. Light blue square denotes disrupted area. Dark blue square denotes intact area. Scale: x = 319.45um, y = 319.45um, z = 36um; Right: Optical cross-section with K14 staining. Scale bar = 50um. Light blue square denotes disrupted area. Dark blue square denotes intact area. Quantification in (H). **(H)** Paired quantification of absolute number of K14^+^ GFP^+^ luminal cells in focally disrupted versus intact portions of the same hyperalveolar lesion. N=3-5 mice.

Finally, we determined how the disruption of the basal-myoepithelial layer correlates with luminal cell-intrinsic features that relate to premalignant progression. Because broad basal disruption was infrequent, focal and broad disruption categories were combined for subsequent analyses. We quantified GFP^+^ cell size, cell circularity, proliferation, and K14 misexpression in hyperalveolar lesions with or without disrupted basal architecture. Interestingly, GFP^+^ cell size, cell circularity, and proliferation were comparable between lesions with or without disrupted basal architecture (Figs. 4A-D), suggesting that increased proliferative activity and pronounced morphological abnormalities within the luminal compartment happen independently of basal disruption. In contrast, K14-misexpressed GFP^+^ luminal cells were significantly more abundant in a subset of hyperalveolar lesions with disrupted GFP^−^ basal architecture (Figs. 4E,F). While K14^+^ GFP^+^ premalignant cells occur at a low frequency in lesions with intact basal architecture, this increase implies a propensity for cell plasticity in response to basal layer disruption.

We next asked whether there is a spatial correlation between basal disruption and luminal cell plasticity. Taking advantage of focal disruption lesions that contain both intact and disrupted areas, we quantified the number of K14^+^ GFP^+^ cells near the intact or focally disrupted areas within the same hyperalveolar lesions and noticed an enrichment in the number of K14^+^ premalignant cells adjacent to the focally disrupted regions of the basal layer (Figs. 4G,H). This finding implies a relationship between basal disruption and K14 misexpression in mutant luminal cells: either luminal cell plasticity is promoted in response to basal layer disruption at a focal level, or luminal cell plasticity promotes basal disruption. The former could be caused by altered environmental cues that luminal cells are now exposed to upon the disruption of basal cell organization [42], while the latter could be caused by altered paracrine signals from K14-misexpressed mutant luminal cells leading to the loss of basal layer integrity [43]. Additionally, these processes could act in a feedforward fashion to mutually promote each other. Taken together, among all luminal features examined, K14 abundance was the only feature significantly correlated with basal architecture disruption, uniquely associating basal layer disruption with luminal cell plasticity.

## Discussion

In this study, we define key phases and tissue architecture alterations during premalignant progression in the MADM-BLBC mouse model. Although most *Brca1,Trp53*-mutant lesions remained as simple ducts, a rare subset progressed to the hyperalveolar phase, where hallmark features of basal-like breast cancer—including increased proliferation, altered cell morphology, and acquisition of basal cytokeratin expression—first emerged. Within this rare population, only a fraction exhibited disruption of the surrounding basal-myoepithelial architecture. Notably, basal architecture disruption uniquely associated with luminal K14 misexpression, but not with increased lesion size, proliferation, or pleomorphic morphology, identifying disruption of the basal-myoepithelial layer as a previously unrecognized feature of premalignant progression that accompanies luminal cell plasticity.

To address the dynamic relationship between the basal-myoepithelial cell layer and premalignant luminal cells, we combined a lineage tracing tool with whole mount clearing, staining and imaging of the mammary fat pad. Our specific genetic mosaic mouse model, MADM-BLBC, has several advantages. First, it mimics human cancer initiation by generating rare mutant cells in a mosaic fashion [33, 44]. Secondly, it phenocopies features found in BRCA carrier disease-free tissue, such as increased proliferation and basal cytokeratin misexpression [30, 45, 46], making it an ideal model for studying premalignant progression of BLBC. Finally, because the MADM system is modular, it can be modified to investigate other aspects of mammary gland biology beyond breast cancer at high spatial resolution *in vivo*. Coupling this model with tissue clearing techniques applicable for immunofluorescence staining of the mammary fat pad [29, 31, 47, 48] enabled high-throughput analyses of spatial interactions between GFP^+^ premalignant luminal cells and colorless, bystanding basal-myoepithelial cells, which would be challenging to resolve using traditional histological techniques.

One central finding in this study was the identification of basal-architecture disruption before malignant transformation, arising in a subset of hyperalveolar lesions. Geometric constraints, previously discovered by model prediction of how premalignant ductal cells should expand over time, are a tissue protection mechanism that limit clonal expansion [29]. While the modeling study did not specifically address the source of geometric constraints, our data suggests that basal-myoepithelial cells may be the cellular mediators of this geometric constraint: while they remain intact in the majority of hyperalveolar lesions to limit their progression, their focal disruptions may release such constraint to permit further progression. However, it remains to be determined the cause of basal layer disruption, as we found it is not simply a result of lesion growth. It is critical to clarify if basal-myoepithelial cells are lost due to cell death, creating gaps in the basal-myoepithelial layer, or have faulty adherens and gap junctions to neighboring basal and luminal cells [21, 43, 49].

We expanded upon our initial observation of basal layer disruption and found a previously unappreciated correlation with luminal cell state plasticity. Among all of the features tested in this study, only K14 misexpression in luminal cells increased with basal disruption. This suggests that increased proliferation and altered cell morphology can occur in premalignant luminal cells even under basal layer constraint, and they are not significantly altered by basal layer disruption. While these features well attributed to cancer grade and stage [26], alone, they may not be indicative of the most high risk precancerous lesions. The correlation between basal disruption and K14 misexpression in the luminal compartment uniquely associates tissue architecture with luminal cell-state plasticity. Lineage plasticity is known to be activated in the mammary gland by several inciting events, including oncogenic insult and environmental factors [35, 36, 38, 42, 50-53]. Our data shows that luminal premalignant cells already have stochastic plasticity at the hyperalveolar phase. However, upon basal architecture disruption, premalignant luminal cells are more prone to K14 misexpression near the site of disruption, suggesting a combined effect of both intrinsic oncogenic changes and extrinsic environmental cues. Future studies are warranted to determine how basal disruption impacts luminal lineage plasticity, or vice versa, thereby paving a way forward to pinpoint action sites for cancer prevention.

One limitation of this study is that individual lesions could not be followed longitudinally to determine whether hyperalveolar lesions with disrupted basal architecture are those that ultimately transform. Furthermore, although basal disruption may represent an early hallmark of progression, its clinical utility will likely depend on identifying secreted biomarkers associated with this tissue architectural transition. Such studies may provide new opportunities for early detection and prevention of BRCA1-associated breast cancer.

## Methods

### Animals

Briefly, stocks used to produce MADM-BLBC and MADM-WT were generated from the following mouse lines, as described in Zeng et. al. 2023: TG11ML (RRID:IMSR_JAX:022977), Brca1flox (RRID:MGI:3618507), p53KO (RRID:IMSR_JAX:002101), GT11ML (RRID:IMSR_JAX:022976) and MMTV-Cre (RRID:IMSR_JAX:003553). The resulting TG11ML,Brca1flox,p53KO stocks and GT11ML; MMTV-Cre stocks were backcrossed to FVB/NJ mice (RRID:IMSR_JAX:001800) for 6 generations and verified using speed congenics (RRID:SCR_009757) to reach > 96% FVB similarity. All animal work was performed in the University of Virginia Animal Vivarium. All procedures, including housing and husbandry were approved by the Institutional Animal Care and Use Committee (IACUC) at the University of Virginia, following national guidelines to ensure the humanity of all animal experiments.

### Genotyping

Genotyping was performed as described in Zeng et. al. 2023. Briefly, the mouse toe was used to extract DNA for PCR. First, 120 μl of 50 mM NaOH was added to each toe and then incubated at 95°C for 20 min in the PCR machine, followed by addition of 30 μl of 1 M Tris-HCl (pH 7.4) and mixing. One microliter of the toe solution was used for the PCR template in a 20 μl PCR reaction. PCR primer sequences were as follows. (1) MADM TG/GT cassettes: primer-1, 5-TGGAGGAGGACAAACTGGTCAC-3; primer-2, 5-TCAATGGGCGGGGGTCGTT-3; primer-3, 5-TTCCCTTTCTGCTTCATCTTGC-3; PCR products, knock-in (KI) band, 230 bp and wild-type band, 350 bp. (2) MMTV-Cre: primer-1, 5-CACCCTGTTACGTATAGCCG-3; primer-2, 5-GAGTCATCCTTAGCGCCGTA-3; PCR product, KI band, 300 bp. (3) p53KO: primer-1, 5-ACCGCTATCAGGACATAGCGTT-GG-3; primer-2, 5-CACAGCGTGGTGGTACCTTATG-3; primer-3, 5-GGTATACTCAGAGCCGGCCTG-3; PCR products, KI band, 700 bp and wild-type band, 450 bp. (4) Brca1flox: primer-1, 5-CTGGGTAGTTTGTAAGCATCC-3, primer-2, 5-TCTTA-GCCCTCAGAAAACTC-3; PCR products, flox/flox band, 365 bp and wild-type band, 297 bp.

### Whole-mount immunofluorescence staining of mammary glands

Whole-mount immunofluorescence staining was adapted from Ciwinska et. al. 2023 and Hannezo & Scheele 2023). The first, second and third mammary glands were dissected and incubated in a mixture of 2.5mg/mL collagenase type I (Thermo Fisher Scientific, Cat#: 17-100-017) and 10ug/mL hyaluronidase (Sigma-Aldrich, Product#: H3884) at 37°C for 20 minutes at 200 rpm, fixed in 4% PFA for 2 hours at room temperature, and incubated for at least 3 hours in blocking buffer containing 1% bovine serum albumin, 5% normal goat serum, 0.8% Triton X-100 and 0.01% sodium azide in PBS. Primary antibodies were diluted in blocking buffer and incubated overnight at 4°C. Glands were optically cleared using a supersaturated solution of 100g sucrose in 50mL ddH20 overnight at room temperature before being mounted on a 25x75x1mm microscopy slide (VWR, Cat#: 48311-703) with a 22x50mm no. 1 coverslip (VWR, Cat#: 48393-059) using VectaShield Antifade mounting media (Vector Laboratories, Ref#: H1900). Another slide is used to firmly press and flatten the mammary gland before sealing with nail polish. Primary antibody used: Keratin 14 polyclonal antibody (1:100; BioLegend, Cat#: 905301, RRID:AB_2565048). Secondary antibodies used: Donkey anti-rabbit IgG AF555 (1:250; Jackson ImmunoResearch Labs Cat# 711-565-152, RRID:AB_3095471), Donkey anti-rabbit IgG AF647 (1:250; Jackson ImmunoResearch Labs Cat# 711-605-152, RRID:AB_2492288). Conjugated primary antibodies used: anti-E-Cadherin-BV421 (1:200; BioLegend, Cat#: 147319, RRID:AB_2750483), anti-Ki67-AF594 (1:200; Thermo Fisher Scientific, Cat#: 755-5698-94, RRID:AB_3679245), anti-Ki67-eFluor660 (1:200; Thermo Fisher Scientific, Cat#: 50-5698-82, RRID:AB_2574235), anti-α Smooth Muscle Actin (SMA)-AF647 (1:200; BioLegend, Cat#: 614856, RRID:AB_3662309).

### Whole-mount imaging of mammary glands

Imaging of whole-mount mammary glands was performed using an inverted Zeiss LSM 900 confocal microscope, equipped with 405nm, 488nm, 561nm and 640nm lasers. Different fluorophores were excited as follows: BrilliantViolet-421 at 405nm, green fluorescent protein (GFP) at 488 nm, red fluorescent protein (RFP) and AlexaFluor-594 at 561 nm, and AlexaFluor-647 and eFluor-660 at 633 nm. BrilliantViolet-421 was collected at 410–470 nm, GFP was collected at 400–632 nm, RFP and AlexaFluor-594 at 400–650 nm, and AlexaFluor-647 and eFluor-660 at 636–700 nm. Whole mount images were acquired with an EC Plan-Neofluar 5x/0.16 DIC objective (Zeiss, Item#: 420331-9911-000) using a Z-step size of 30um (total Z-stack around 90um) spanning large tile-scan areas. Higher magnification tiled images were acquired with a Plan-Apochromat 10x/0.45 M27 objective (Zeiss, Item#: 420640-9900-000) using a Z-step size of 4um (total Z-stack around 36um) spanning 25 tiles. Detailed images of individual phases were obtained with a Plan-Apochromat 20x/0.8 M27 objective (Zeiss, Item#: 420650-9902-000) using a Z-step size of 4um (total Z-stack around 36um). All images were stitched and processed using ZEN 3.4 and further processed using IMARIS software.

### Whole-mount image processing

Using Imaris 11.0, a surface was created using the Machine Learning module to mask and measure contiguous GFP^+^ lesions in whole-mount images (**Supp 1D**). Data tables from Imaris were imported into R studio or GraphPad Prism to compose graphs. Single-cell segmentation masks of luminal epithelial cells were generated from individual ECAD z-stack immunofluorescence images using CellPose-SAM (CellPose v4.0.5). The resulting masks were imported into CellProfiler for downstream single-cell feature extraction, such as cell size, cell circularity, Ki67-positivity or K14-positivity. Data tables from CellProfiler were imported into R studio or GraphPad Prism to compose graphs.

### Statistics

Statistical analysis was performed with GraphPad Prism. The normality of data distribution was checked with qqplot in R. P values and statistical tests performed are included in the figure legends.

## Supplementary Material

This is a list of supplementary files associated with this preprint. Click to download.

• floatimage1.png
